# Prognostic significance of high mobility group A2 (HMGA2) in pancreatic ductal adenocarcinoma: malignant functions of cytoplasmic HMGA2 expression

**DOI:** 10.1007/s00432-021-03745-w

**Published:** 2021-07-24

**Authors:** Jan-Paul Gundlach, Charlotte Hauser, Franka Maria Schlegel, Anna Willms, Christine Halske, Christian Röder, Sandra Krüger, Christoph Röcken, Thomas Becker, Holger Kalthoff, Anna Trauzold

**Affiliations:** 1 Department of General Surgery, Visceral-, Thoracic-, Transplantation- and Pediatric-Surgery, University Hospital Schleswig-Holstein (UKSH), Campus Kiel, Arnold-Heller-Str. 3, Building C, 24105 Kiel, Germany; 2 Institute for Experimental Cancer Research, University of Kiel, Arnold-Heller-Str. 3, Building U30, 24105 Kiel, Germany; 3 Department of Pathology, UKSH, Campus Kiel, Arnold-Heller-Str. 3, Building U33, 24105 Kiel, Germany

**Keywords:** HMGA2, Prognosis, Immunohistology, Pancreatic cancer, Nucleus

## Abstract

**Purpose:**

HMGA2 has frequently been found in benign as well as malignant tumors and a significant association between HMGA2 overexpression and poor survival in different malignancies was described. In pancreatic ductal adenocarcinoma (PDAC), nuclear HMGA2 expression is associated with tumor dedifferentiation and presence of lymph node metastasis. Nevertheless, the impact of HMGA2 occurrence in other cell compartments is unknown.

**Methods:**

Intracellular distribution of HMGA2 was analyzed in PDAC (*n* = 106) and peritumoral, non-malignant ducts (*n* = 28) by immunohistochemistry. Findings were correlated with clinico-pathological data. Additionally, intracellular HMGA2 presence was studied by Western blotting of cytoplasmic and nuclear fractions of cultured cells.

**Results:**

HMGA2 was found in the cytoplasm and in the nucleus of cultured cells. In human tumor tissue, HMGA2 was also frequently found in the cytoplasm and the nucleus of tumor cells, however, nuclear staining was generally stronger. Direct comparison from tumor tissue with corresponding non-neoplastic peritumoral tissue revealed significantly stronger expression in tumors (*p* = 0.003). Of note, the nuclear staining was significantly stronger in lymph node metastatic cell nuclei compared to primary tumor cell nuclei (*p* = 0.049). Interestingly, cytoplasmic staining positively correlated with lymph vessel (*p* = 0.004) and venous invasion (*p* = 0.046).

**Conclusion:**

HMGA2 is a prognostic marker in PDAC. Firstly, we found a positive correlation for cytoplasmic HMGA2 expression with lympho-vascular invasion and, secondly, we found a significantly stronger nuclear expression of HMGA2 in cancer-positive lymph node nuclei compared to primary tumor cell nuclei. So far, the role of cytoplasmic HMGA2 is nearly unknown, however, our data lend support to the hypothesis that cytoplasmic HMGA2 expression is involved in nodal spread.

## Introduction

Effective treatment of patients with pancreatic ductal adenocarcinoma (PDAC) requires early diagnosis and intervention. Although considerable efforts have been made to identify underlying molecular mechanism and novel sensitive specific tumor biomarkers, PDAC still remains one of the deadliest cancers with a mortality rate almost equal to its incidence rate (Siegel et al. 2020). Identification of reliable and reproducible biomarkers would enable better stratification of patients, and eventually provide a guide for individualized therapy. The high mobility group A2 (HMGA2/HMGI-C) is an architectural transcription factor and belongs to the high mobility group AT-hook (HMGA) gene family. It is highly expressed in embryonic tissue, whereas its expression drops during the differentiation being hardly detectable in healthy adult tissue (Chiappetta et al. 1996; Huang et al. 2018). Interestingly, HMGA2 is re-expressed and becomes again highly elevated in benign (Dreux et al. 2010; Tallini et al. 2000) as well as malignant neoplasms such as ovarian cancer (Wu and Wei 2013; Xi et al. 2014; Jin et al. 2018), breast cancer (Wu et al. 2016; Sgarra et al. 2018), lung cancer (Kumar et al. 2014), gastrointestinal cancer (Mito et al. 2017; Zhu et al. 2017; Huang et al. 2018; Wang et al. 2011; Zhang et al. 2016), and pancreatic cancer (Strell et al. 2017; Piscuoglio et al. 2012). Importantly, diverse meta-analyses revealed a correlation of high HMGA2 expression with poor patient’s survival in various malignancies such as gastric, colorectal as well as head-and-neck cancers (Binabaj et al. 2019; Nie et al. 2018; Huang et al. 2018). For hepatobiliary cancers, in particular HCC, cholangiocarcinoma and gallbladder cancer (Binabaj et al. 2019; Nie et al. 2018; Huang et al. 2018) as well as PDAC (Huang et al. 2018; Binabaj et al. 2019), poor survival was reported. Of note, not all tumors with elevated HMGA2 expression show significant association with survival rates (e.g., ovarian cancer Huang et al. 2018; Nie et al. 2018) or esophageal cancer (Huang et al. 2018). Thus, HMGA2 represents a reliable marker of prognostic value in some, but not all cancers.

Analyses of the HMGA2 expression in normal pancreatic tissue and pancreatic cancer revealed clearly elevated levels in the latter with significant association with malignancy (Hristov et al. 2009; Piscuoglio et al. 2012; Strell et al. 2017; Li et al. 2020). An increasing HMGA2 expression along with the PDAC development from normal pancreatic tissue, intraepithelial neoplasms (PanIN), and PDAC was detected (Piscuoglio et al. 2012; Strell et al. 2017) suggesting again its malignancy enhancing potential. In accordance, HMGA2 expression showed a positive correlation with tumor grade and progression: expression of HMGA2 increases upon dedifferentiation tumors (Hristov et al. 2009; Piscuoglio et al. 2012; Strell et al. 2017; Gong et al. 2019) and with the presence of lymph node metastases (Hristov et al. 2009; Piscuoglio et al. 2012; Gong et al. 2019; Li et al. 2020). Within recent studies, overall survival was found significantly associated with the level of HMGA2 expression (Haselmann et al. 2014; Strell et al. 2017; Gong et al. 2019; Li et al. 2020).

One of the known mechanisms underlying the pro-tumoral functions of HMGA2 is its role in the induction of epithelial–mesenchymal transition (EMT), a process linked to the acquisition of metastatic capability of tumor cells. Here, acting in its canonical way as a transcriptional regulator, HMGA2 enhances the expression of EMT regulators like Snail, Twist, Slug and ZEB1 thereby down regulating the levels of E-cadherin and upregulating vimentin (Thuault et al. 2008; Sgarra et al. 2018). Interestingly, Morishita et al*.* reported that overexpression of HMGA2 converted nonmetastatic 4TO7 breast cancer cells to metastatic cells that homed specifically to the liver in a mouse allograft model (Morishita et al. 2013). Of note, expression of HMGA2 is known to enhance different signaling pathways such as TGFβ signaling (Kou et al. 2018) which has been linked to metastasis (Xie et al. 2018). In addition, HMGA2 can induce EMT via MAPK (Watanabe et al. 2009; Hawsawi et al. 2018) and via the Wnt/β-catenin pathway (Zha et al. 2013). In accordance, HMGA2 smoothens the way for a metastatic phenotype and EMT in renal cell carcinoma (Kou et al. 2018), PDAC (Watanabe et al. 2009; Gong et al. 2019) and gastric cancer (Zha et al. 2013). Altogether, these and other data disclosed the role of HMGA2 as a key regulator of EMT and one of the major players in establishing a malignant phenotype in different tumors of epithelial origin, including pancreatic cancer.

Importantly, in the vast majority of histochemical studies analyzing the relevance of HMGA2 for cancer development, progression and disease outcome, a solely nuclear presence of this protein was analyzed. However, our recent report on HMGA2 in breast cancer clearly revealed a prognostic significance of cytoplasmic HMGA2. In particular, high levels of cytoplasmatic HMGA2 were associated with a favorable overall survival of breast cancer patients (Heilmann et al. 2020). In detail, HMGA2 expression was linked to better survival in triple negative breast cancer and well-differentiated estrogen receptor-positive breast cancer patients irrespective of lymph node metastases or tumor size. To the best of our knowledge, no comparable data are available so far for PDAC. To fill this gap of information, we tested in the present study the hypothesis that cytoplasmic expression of HMGA2 also impacts the malignant phenotype of PDAC.

## Methods

### Cell culture

The pancreatic cancer cell lines Panc1, Panc89, BxPC3 and colon carcinoma cell line HCT116 were cultured in RPMI 1640 supplemented with 10% FCS, 2 mM glutamine and 1 mM sodium pyruvate (all from Life Technologies Inc., Karlsruhe, Germany). For the preparation of nuclear and cytoplasmic cell extracts cells were grown for 24 h in 6-well plates and the NE-PER™ nuclear and cytoplasmic extraction reagents (Thermo Fischer Scientific, Darmstadt, Germany) were used according to the manufacturer’s protocol.

### Western blot analysis

Nuclear and cytoplasmic cell fractions were separated on 4–20% Tris–Glycine gels (Invitrogen, Thermo Fisher Scientific, USA), blotted on PVDF-membrane and incubated with the appropriate primary antibody followed by incubation with the HRP-conjugated secondary antibody (Cell Signaling, Frankfurt, Germany). Antigen visualization was performed by enhanced chemiluminescence (ECL-kit, Amersham Pharmacia Biotech, England). Primary antibodies against HMGA2, α-tubulin, and lamin A/C (all rabbit) were purchased from Cell Signaling (Frankfurt, Germany).

### Study cohort

For this study, formalin-fixed and paraffin-embedded PDAC and adjacent, peritumoral non-malignant tissue samples were used. Probes were retrieved form the archive of the Dept. of Pathology of the University Hospital Schleswig-Holstein and Christian-Albrechts-University Kiel spanning the period from 1999 to 2010. Follow-up data were obtained from the Epidemiological Cancer Registry Schleswig-Holstein, Germany and hospital records. Only patients with an adenocarcinoma of the pancreas were included. pTNM category was determined according to the 8th edition of the Union for International Cancer Control (UICC) guidelines (Brierley et al. 2016). Approval for this study was granted by the local institutional review board of the Medical Faculty of the Christian-Albrechts-University of Kiel (A-110/99).

### Immunohistochemistry

Serial 3 µm paraffin sections were deparaffinized and rehydrated with xylene and rehydrated in a descending alcohol series. Antigen retrieval was done with citrate-buffer (pH 6.0) for 15 min at 120 °C, followed by blocking of endogenous peroxidase-activity with Hydrogen-Peroxide Block [15 min, room temperature (RT); Thermo Scientific, Fremont, CA]. Slides were incubated with primary antibody antibody (HMGI-C S-15) 1:50 (20 µg/ml) (Santa Cruz Biotechnology, Dallas, Texas) 1:50 (20 µg/ml) diluted in antibody diluent for 2 h at RT. Bound antibodies were visualized with the Histofine polymer (Histofine Simple Stain MAX PO Immuno-peroxidase Polymer Anti-Goat, Nichirei Biosciences, Tokyo, Japan) and diaminobenzidine (DAB Peroxidase Substrate Kit, Vector Laboratories, Burlingham, California). All slides were counterstained with hemalum and cover slipped.

### Histopathological scoring

For evaluation of the staining, a two-dimensional scoring system was applied to semi-quantitatively assess the HMGA2 expression data on a Leica DM 1000-Microscope (Leica, Wetzlar, Germany) as described earlier (Gundlach et al. 2018). The intensity of the staining was defined on an arbitrary scale of 0–3 with 0: no staining; 1: weak staining; 2: moderate staining and 3: strong staining. In case of varying staining intensities, strongest values were recorded. Additionally, the percentage of stained cells was quantified and scaled from 0 to 4 with 0: no positive cells; 1: 1–10%; 2: 10–50%; 3: 51–80%; and 4: 81–100% positively stained cells. After being separately assessed for cytoplasm and nuclei by two independent pathologists, the values were summarized in a sum score as follows (Table 1): the addition of intensity and quantity scoring resulted in an immunoreactivity sum score. The sum score ranged from 0 to 7 for nuclear and cytoplasmatic immunoreactivity, respectively.

**Table 1:** Histomorphological evaluation score

Staining intensity	Points	Number of positive cells	Points
Negative	0	0%	0
Weakly positive	1	< 10%	1
Moderately positive	2	10–50%	2
Strongly positive	3	51–80%	3
–	–	81–100%	4

### Statistical analyses

Statistical analyses were performed with SPSS 25.0 (SPSS, IBM Corporation, Armonk, NY, USA). Correlation of clinico-pathological patient characteristics and HMGA2 expression was conducted by dichotomization and appliance of Kendall’s Tau (*τ*) test. We included only patients with existing follow-up data, whereas patients who died within 14 days after surgery as well as patients who received neoadjuvant treatment were excluded. For these analyses 97 out of 106 patients were included, whereof, 19 patients were censored because they were either alive or lost in follow-up. We analyzed the overall postoperative survival. Evaluation of normal and malignant tissue staining intensities was performed with the Wilcoxon test as a nonparametric test for paired samples. Survival analyses were performed by Kaplan–Meier estimates with subsequent statistical evaluation by log-rank tests. *p* values ≤ 0.05 were considered significant.

## Results

### HMGA2 is localized to the cytosol and nucleus in different tumor cell lines

**Fig. 1:**
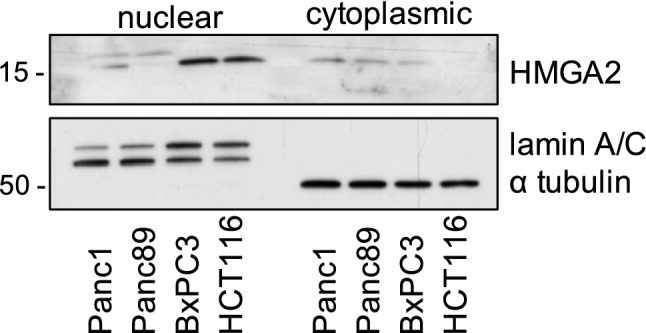
HMGA2 is present in the cytosol and nucleus in different tumor cell lines. Western blotting of nuclear and cytoplasmic extracts of three PDAC cell lines Panc1, Panc89, BxPC3 and colon carcinoma cells HCT116. Loading control is represented with staining against lamin A/C and α-tubulin

In the present study, we aimed to investigate whether the expression of HMGA2 and, in particular, the pattern of its intracellular distribution correlates with histopathological parameters and correlates with patient prognosis. To exclude the possibility of false-positive cytoplasmic immunostaining, we additionally performed Western blot analyses of cytosolic and nuclear extracts of three PDAC cell lines, i.e., Panc1, Panc89 and BxPC3, as well as the colon carcinoma cells HCT116. The results shown in Fig. 1 confirmed the presence of HMGA2 in the nuclei as well as in the cytoplasmic compartments in all four cell lines. In particular, for Panc1 and Panc89 cell lines, comparable expression was detected in cytosolic and nuclear fractions, while HMGA2 was more present in the nuclear fraction of BxPC3 cells and in the colon cancer cell line HCT116.

### Patient cohort

In order to evaluate the staining intensity, percentage of stained cells and intracellular distribution of HMGA2 in sections of 106 tumors and 28 neighboring histological normal pancreatic ducts from 106 PDAC patients were analyzed as described before (Gundlach et al. 2018). Thereof, 51 (48.1%) patients were female and the median age of the whole cohort was 65 years (range 47–85 years). The anatomical location of the tumor was in the pancreatic head in 75/106 (70.8%), in the corpus in 7/106 (6.6%), and in the tail in 8/106 (7.5%) of the cases. No specification was stated in 16/106 cases (15.1%). We provide detailed clinico-pathological patient characteristics in Table 2. Almost 90 percent of the patients have undergone surgery at category T3 (94/106; 88.7%) with existing lymph node metastases (84/106; 79.2%). No patient was operated at category T1. Resected tumors were well or moderately differentiated in two-thirds (66.1%) of the cases. Distant metastasis was only present in 10.4% of the cases (11/106).

**Table 2:** Clinico-pathological patient characteristics on the basis of the TNM status (according to the UICC Classification of Malignant Tumors)

Feature	*n*	%
T—tumor category		
T1	0	0.0
T2	3	2.8
T3	94	88.7
T4	9	8.5
N—nodal spread		
N0	22	20.8
N1	84	79.2
NX	0	0.0
M—distant metastasis		
M0	69	65.1
M1	11	10.4
MX	26	24.5
Venous invasion		
V0	79	74.5
V1	19	17.9
V2	3	2.8
VX	5	4.7
Perineural invasion		
Pn0	39	36.8
Pn1	59	55.7
PnX	8	7.5
Lymphatic invasion		
L0	30	28.3
L1	71	67.0
LX	5	4.7
R—status		
R0	74	69.8
R1	28	26.4
R2	2	1.9
RX	2	1.9
Histopathological grading		
G1	11	10.4
G2	59	55.7
G3	35	33.0
G4	1	0.9

Given are the total number of patients and the percentage (%)

T1: tumor < 2 cm; T2: > 2 < 4 cm, T3: > 4 cm, T4: tumor involves coeliac axis, superior mesenteric artery and/or common hepatic artery

### Expression of HMGA2 in PDAC and non-malignant adjacent tissue

**Fig. 2:**
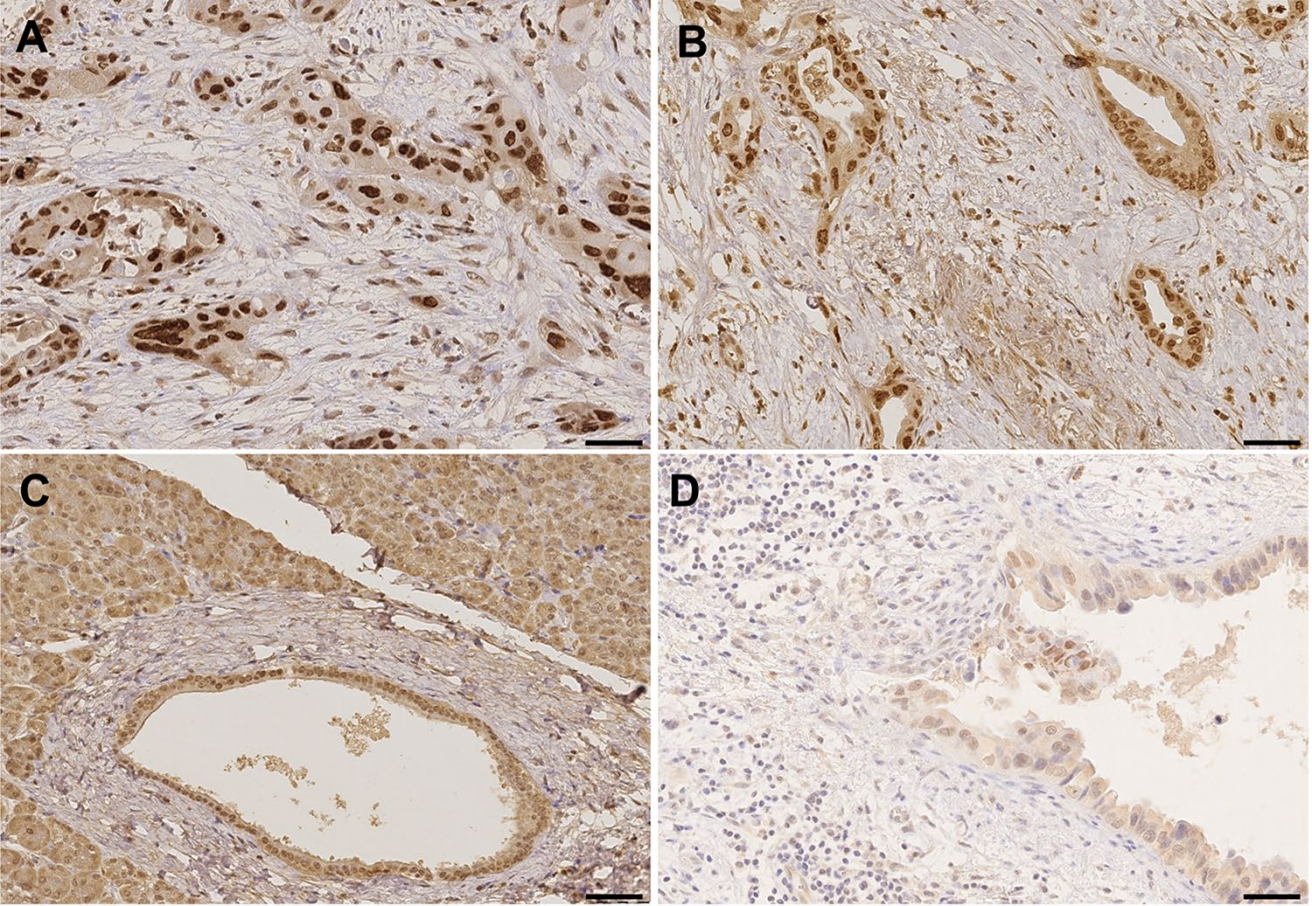
Representative images of HMGA2 staining in PDAC tissue (**A**, **B**), non-neoplastic pancreatic duct (**C**) and lymph node metastasis (**D**). **A** Tumors with strong nuclear staining in > 80% cells without cytoplasm staining; **B** tumors with cytoplasm and nuclear staining; **C** non-neoplastic duct with weak to moderate positive cytoplasm staining and with moderate to strong nuclei staining; **D** lymph node metastasis with strong nuclear staining. Scale bar marks 50 µm (**A**, **B**, **D**) as well as 100 µm (**C**)

HMGA2 was frequently found in tumor cells (Table 3). Representative images showing expression pattern of HMGA2 in tumor tissue and non-malignant, adjacent tissue are displayed in Fig. 2. In 86.8% (92/106), positive cytoplasmic staining and in 98.1% (104/106), positive nuclear staining was detected. Figure 2A + B represents tumors with nuclear staining without (A) and with simultaneous cytoplasm staining (B). In 76.4% (81/106) and 79.2% (84/106) of the cases, more than every second nucleus and every second cytoplasm was positively stained, respectively. Interestingly, the nuclear staining was in general stronger than the cytoplasmic staining (58.5% (62/106) moderate or strong in the nucleus vs. 3.8% (4/106) moderate or strong in the cytoplasm).

**Table 3:** Cytoplasmic and nuclear HMGA2 expression in malignant and non-malignant ducts

(a)	Cytoplasm		Nuclei	
	*n*	%	*n*	%
Positive tumor cells				
0%	14	13.2	2	1.9
< 10%	0	0.0	2	1.9
11—50%	8	7.5	21	19.8
51—80%	22	20.8	22	20.8
> 80%	62	58.5	59	55.7
Staining intensity				
Negative	14	13.2	2	1.9
Weakly positive	88	83.0	42	39.6
Moderately positive	4	3.8	35	33.0
Strongly positive > 80%	0	0.0	27	25.5
Sum score				
0	14	13.2	2	1.9
2	0	0.0	1	0.9
3–4	30	28.3	25	23.6
5–6	62	58.5	57	53.7
7	0	0.0	21	19.8

(b)	Cytoplasm				Nuclei			
	Tumor		Non-malignant		Tumor		Non-malignant	
Staining intensity	*n*	%	*n*	%	*n*	%	*n*	%
Negative	5	17.9	8	28.6	1	3.6	5	17.9
Weakly positive	23	82.1	18	64.3	13	46.4	14	50.0
Moderately positive	0	0.0	2	7.1	7	25.0	9	32.1
Strongly positive	0	0.0	0	0.0	7	25.0	0	0.0
Staining pattern: tumor vs. non-malignant	No difference ns, *p* = 0.5				** *p* = 0.003			

(a) number of positive cells, staining intensity and corresponding sum score are shown for cytoplasm and nucleus separately. Additionally, particular staining intensity in relation to histologic specification (tumor vs. peritumoral non-malignant) and for cytoplasmic vs. nuclear staining are provided in (b)

HMGA2 was more frequently found both in the cytoplasm and the nuclei of tumor cells than in normal duct cells [Fig. 2C; cytoplasm 23/28 (82.1%) vs. 20/28 (71.4%) and nuclei 27/28 (96.4%) vs. 23/28 (82.1%)]. Moreover, cytoplasmic staining of HMGA2 was present with similarly low intensity in tumor and normal tissue (negative to weak positive in 28/28 and 26/28 cases, respectively; *p* = 0.5). In contrast, significant differences were found in the intensity of nuclear staining (negative to weak positive in 14/28 and 19/28 of the cases, respectively; *p* = 0.003), thereof, in 7 cases strong intensity was found in the nuclei of tumor cells. However, we could not detect any kind of mutual exclusion between staining of malignant and non-malignant tissue.

### Expression of HMGA2 in lymph node metastases

In 17 patients, lymph node metastases were analyzed (Fig. 2D). HMGA2 was expressed in 64.7% (11/17) of the cytoplasm and in 94.1% (16/17) of the nuclei. Interestingly, HMGA2 staining was significantly stronger in lymph node nuclei compared to the individual corresponding nuclei of the primary tumor (*p* = 0.049). In contrast, the cytoplasmic presence of HGMA2 was not significantly different, neither in the staining intensity nor in the number of positive cells between the primary tumor and its lymph node metastases.

### Correlation of HMGA2 expression with clinico-pathological parameters and patient survival

Subsequently, we correlated the expression level of HMGA2 and its intracellular distribution (cytoplasm and nucleus) with diverse clinico-pathological parameters (Table 4). A significant positive correlation of the nuclear HMGA2 staining intensity with tumor grading (*τ* = 0.193; *p* = 0.028) was assessed as previously described (Haselmann et al. 2014). Interestingly, the staining intensity of cytoplasmic HMGA2 positively correlated with lymph vessel invasion (*p* = 0.004) and the number of positively stained cells is associated with venous invasion (*p* = 0.046).

**Table 4:** Correlation of HMGA2 expression with clinico-pathological parameters

Staining parameter	Tumor category	Nodal spread	Distant metastasis	Venous invasion	Perineural invasion	Lymph vessel	Grading
Intensity cytoplasm	τ = 0.033 p = 0.731	τ = − 0.003 p = 0.971	τ = − 0.135 p = 0.224	τ = 0.182 p = 0.061	τ = − 0.041 p = 0.680	**τ = 0.285 p = 0.004**	τ = − 0.150 p = 0.105
Number pos. cytoplasm	τ = − 0.020 p = 0.827	τ = 0.094 p = 0.309	τ = 0.052 p = 0.624	**τ = 0.185 p = 0.046**	τ = 0.002 p = 0.980	τ = 0.096 p = 0.309	τ = − 0.120 p = 0.174
Sum score cytoplasm	τ = 0.004 p = 0.961	τ = 0.083 p = 0.359	τ = 0.024 p = 0.818	τ = 0.176 p = 0.056	τ = − 0.007 p = 0.942	τ = 0.119 p = 0.203	τ = − 0.098 p = 0.263
Intensity nuclei	τ = 0.124 p = 0.172	τ = 0.073 p = 0.427	τ = − 0.178 p = 0.092	τ = 0.016 p = 0.865	τ = 0− .065 p = 0.494	τ = − 0.056 p = 0.551	**τ = 0.193 p = 0.028**
Number pos. nuclei	τ = 0.015 p = 0.868	τ = 0.123p = 0.181	τ = 0.006 p = 0.952	τ = 0.064 p = 0.490	τ = 0.143 p = 0.136	τ = − 0.116 p = 0.220	τ = − 0.006 p = 0.948
Sum score nuclei	τ = 0.087 p = 0.316	τ = 0.124 p = 0.157	τ = − 0.125 p = 0.214	τ = 0.047 p = 0.594	τ = 0.049 p = 0.594	τ = − 0.093 p = 0.299	τ = 0.103 p = 0.223

Shown are correlation coefficients Kendall’s *τ* as well as the significance of the correlation

Bold numbers represent significant (*p* ≤ 0.05) results

*τ* Kendall’s *τ*, *p* *p* value

**Fig. 3:**
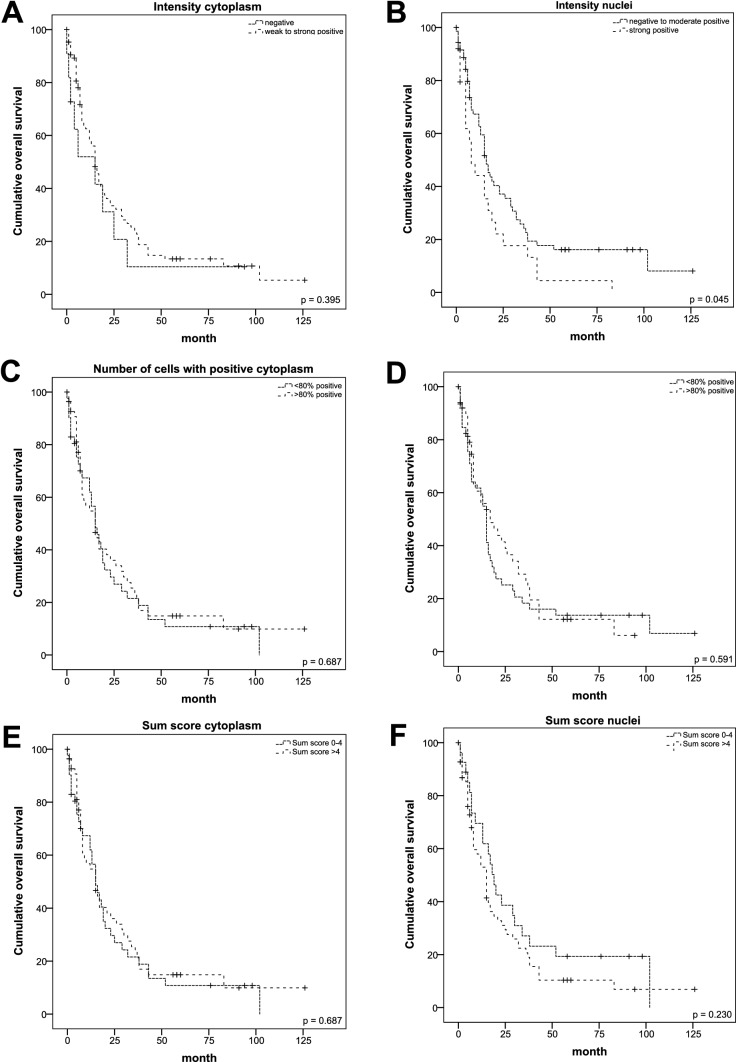
Kaplan–Meier analyses of the cumulative survival of patients with differential expression of HMGA2. **A**, **B** HMGA2 intensity in the cytoplasm (**A**) and the nuclei (**B**). In the cytoplasm, no HMGA2 expression was dichotomized with weak to strong expression. In the nuclei, we dichotomized non to weak expression with moderate to strong expression. Graph **C**, **D** display patient survival in correlation with number of positively stained cells for the cytoplasm (**C**) and the nuclei (**D**), respectively. We compared equal to less than 80% positively stained cells with more than 80% positively stained cells with cytoplasm and nuclear staining, respectively. **E**, **F** Display survival curves for sum score with 0–4 points compared to > 4 points. *p*-values were calculated by the log-rank test and *p* ≤ 0.05 was considered significant

Moreover, we explored whether the HMGA2 expression pattern could be of prognostic value as shown in Table 5. To address this issue, we dichotomized the results for intensity and number of positive cells as well as the sum score in a group with strong and in a group with weak expression of HMGA2 and analyzed these data by Kaplan–Meier analysis (Fig. 3). Cumulative survival was compared by log-rank test and *p* values ≤ 0.05 were considered significant. Patients with a strongly positive expression for nuclear HMGA2 showed a significantly reduced overall survival (8 months vs. 16 months; *p* = 0.045) (Fig. 3B). However, neither the number of cells with positively stained nuclei nor their sum score showed a significant correlation (Fig. 3D + F). Furthermore, for cytoplasmic HMGA2 staining, no significant correlation with patient`s survival was detected. In detail, neither the intensity nor the number of positively stained cells nor the sum score showed a positive correlation (Fig. 3A, C + E).

**Table 5:** Impact of HMGA2 expression pattern on survival of PDAC patient

Staining parameter	Total number of patients	Number of deceased patients	Median survival ± SD (95% CI) in months	*p*-value
Intensity cytoplasm				
Negative	11	9	15 ± 8.268 (0.000–31.205)	
Weakly to strongly positive	86	69	15 ± 1.558 (11.946–18.054)	0.395
Number of cells with positively stained cytoplasm				
≤ 80%	41	35	15 ± 2.403 (10.291–19.709)	
> 80%	56	43	15 ± 3.453 (8.233–21.767)	0.687
Sum score cytoplasm				
≤ 4	41	35	15 ± 2.403 (10.291–19.709)	
> 4	56	43	15 ± 3.453 (8.233–21.767)	0.687
Staining intensity nuclei				
Negative to weakly positive	72	55	16 ± 1.952 (12.174–19.826)	
Moderately to strongly positive	25	23	8 ± 2.359 (3.375–12.625)	**0.045**
Number of cells with positively stained nucleus				
≤ 80%	47	39	15 ± 1.217 (12.614–17.386)	
> 80%	50	39	17 ± 6.635 (5.956–28.044)	0.591
Sum score nuclei				
≤ 4	28	22	19 ± 2.531 (14.039–23.961)	
> 4	69	56	15 ± 1.892 (11.293–18.707)	0.230

*p*-values were estimated by log-rank-test with p ≤ 0.05 considered as significant and represented in bold numbers

*SD* standard deviation, *CI* confidence interval

## Discussion

HMGA2 was attributed as a prognostic marker in PDAC and different cancer types. Its expression is associated with advanced tumor grades, tumor dedifferentiation, lymph node metastases and poor patient prognosis (Hristov et al. 2009; Piscuoglio et al. 2012; Haselmann et al. 2014; Strell et al. 2017; Gong et al. 2019; Li et al. 2020). In the present study, we validated HMGA2 as a prognostic marker which correlates with malignant cell state in PDAC. We found a higher HMGA2 expression in tumor tissue compared with peritumoral tissue. Moreover, nuclear expression of HMGA2 was significantly stronger in lymph node nuclei than primary tumor cell nuclei. Correlation of HMGA2 expression with clinico-pathological parameters revealed a significant correlation of HMGA2 nuclei staining intensity with tumor grading. Moreover, a strong positive HMGA2 nuclei staining was associated with reduced overall survival. Importantly, along with nuclear expression of HMGA2 we also detected distinct cytoplasmic localization of HMGA2 in tumor cells. Cytoplasmic HMGA2 expression was found to positively correlate with lympho-vascular invasion.

Nuclear occurrence of HMGA2 has been known for a long time as it provides several nuclear functions including an involvement in cell cycle process, DNA damage repair, EMT, apoptosis, senescence and telomere restoration. HMGA2 is highly expressed in embryonic tissue whereas its expression is strictly downregulated in adult somatic cells (Chiappetta et al. 1996; Huang et al. 2018). Overexpression of HMGA2 has been attributed to a feature of malignancy. Nevertheless, HMGA2 could be detected in some non-malignant pancreatic ducts but its expression was higher in tumor tissue compared to non-malignant tissue, especially in the nuclei. This characteristic has been described before in PDAC (Abe et al. 2003; Hristov et al. 2009; Piscuoglio et al. 2012; Haselmann et al. 2014; Strell et al. 2017; Gong et al. 2019; Li et al. 2020). Apart from an expression in duct epithelia, we saw a subtle HMGA2 expression in acinar and endocrine cells with no difference in terms of expression pattern or color intensity. For this observation we do not have an explanation so far. This should be clarified in the context of further investigations.

HMGA2 is increasingly expressed in poorly differentiated tumors. In line with other studies, here we report a significant correlation of nuclei HMGA2 staining with tumor grading (Hristov et al. 2009; Piscuoglio et al. 2012; Gong et al. 2019; Strell et al. 2017; Li et al. 2020).

In addition to primary tumors and peritumoral non-malignant tissue, some lymph node metastases were also examined immunohistochemically. The intensity of nuclear staining was significantly higher in lymph node metastases compared to corresponding primary tumors. The number of patients from whom lymph node metastases could be examined was rather low with 17 patients. Hristov et al*.* demonstrated in a larger cohort a significant positive correlation of HMGA2 expression with lymph node metastases (Hristov et al. 2009). A high tumor grade and lymph node metastases are clinico-pathological parameters that are accompanied with worse patient prognosis. Correspondingly, patients with strong positive nuclear HMGA2 expression showed a significantly reduced overall survival (*p* = 0.035) (Strell et al. 2017; Haselmann et al. 2014; Gong et al. 2019).

The role of HMGA2 in EMT and metastatic spread has not yet been fully understood in pancreatic cancer (Gong et al. 2019), although, repeatedly studies have shown that overexpression of HMGA2 is accompanied by a more mesenchymal phenotype in several cancer cells. HMGA2 was described to be responsible in conjunction with the oncogenic RAS signaling pathway for cell growth and EMT in human pancreatic cancer cells (Watanabe et al. 2009). RAS and HMGA2 are known to be translationally downregulated by the let-7 microRNA family, and loss of let-7 expression led to progression of some human cancers (Johnson et al. 2005). Noteworthy, investigations by our group found a connection between TRAIL-R2 and let-7 microRNA. TRAIL-R2 was demonstrated to be located in the nucleus inhibiting the maturation of let-7 microRNA, leading to increased expression of HMGA2 in PDAC cells (Haselmann et al. 2014). In addition, staining intensities of nuclear HMGA2 and TRAIL-R2 showed a significant positive correlation (Haselmann et al. 2014).

In contrast to nuclear HMGA2, cytoplasmic HMGA2 has not been well characterized so far. In our dataset, we encouraged the histochemical cytoplasmic HMGA2 staining by analyzing cytoplasmic and nuclear fractions of three PDAC cell lines as well as a colon cancer cell line. Western blot results revealed an unmistakable detectability of HMGA2 in cytoplasmic cell lysates. Cytoplasmic HMGA2 has been also found by others in various tumor entities (Abe et al. 2003; Rahman et al. 2009; Gong et al. 2019; Heilmann et al. 2020). Noteworthy, yet, to the best of our knowledge, there are no reports analyzing the clinical relevance of the intracellular distribution of HMGA2 in PDAC. Unexpectedly, we found a correlation of cytoplasmic, but not of nuclear HMGA2 staining to lymphatic invasion and venous invasion in PDAC. This suggests that HMGA2 possesses distinct, compartment-dependent pro-tumoral functions.

Heilmann et al. described cytoplasmic HMGA2 as an autonomous phenomenon with a prognostic effect in breast cancer patients. High levels of cytoplasmic HMGA2 were associated with a favorable overall survival of breast cancer patients (Heilmann et al. 2020). However, the mechanism of how cytoplasmic HMGA2 favors patient survival needs further investigations. Although the extranuclear localization has long been recognized, little is known about possible functions of HMGA family proteins in this localization.

HMGA1 was described to translocate during late S- and G2-phase from the nucleus to the mitochondria (Nissen et al. 1991; Reeves et al. 1991; Dement et al. 2005). This movement is reported to be very dynamic, bidirectional and cell-cycle dependent. Furthermore, post-translational phosphorylation of HMGA1 proteins by cdc2 kinase alters the binding capacity of HMGA1 for DNA (Reeves et al. 1991) and favors its translocation. Nevertheless, in the mitochondria HMGA1 can bind to mitochondrial DNA (mtDNA) at the D-loop control region (Dement et al. 2005) and through this impacts on mitochondrial DNA maintenance and organelle functions (Dement et al. 2007). This transporting from the nucleus to the cytoplasm is deregulated in cancer cells (Dement et al. 2005). Interestingly, in malignant cells, HMGA1 is reported to be present in the cytoplasm throughout all stages of the cell cycle (Dement et al. 2007). In addition to mtDNA binding, here, HMGA1 inhibits p53-mediated apoptosis by blocking the binding of p53 to the anti-apoptotic factor Bcl-2 (Esposito et al. 2012). In addition to HMGA1, high mobility box 1 (HMGB1) proteins were also described in an extranuclear localization in the cytosol and mitochondria. Along with the receptor for advanced glycation end products, HMGB1 was described to enhance mitochondrial ATP production in malignant cells contributing to tumor progression (Kang et al. 2013). Nevertheless, cytoplasmic functions of HMGA2 have not been unraveled till now.

The prognostic impact especially of parameters like venous and lymphatic invasion is impeded by the overall bad prognosis and high lethality (due to other characteristics) of PDAC. Consequently, a possible prognostic impact of cytoplasmic HMGA2 staining intensity should be analyzed in other tumor entities with a better prognosis (for instance, early stage colorectal carcinoma) to fully evaluate the value of HMGA2 staining as a biomarker for better stratification of cancer patients, e.g., for adjuvant therapy.

## Conclusion

In summary, our data indicate that HMGA2 might possess distinct, compartment-dependent pro-tumoral functions in PDAC. Not only nuclear, but also cytoplasmic expression of HMGA2 indicates a malignant phenotype. Interestingly, cytoplasmic HMGA2 significantly correlated with lymphatic and venous invasion. Thus, our data point to the necessity to investigate the unknown biological functions of cytoplasmic HMGA2.

## Data Availability

The clinical datasets supporting the conclusions of this study were derived from the patient files (paper and electronic form). Therefore, restrictions to availability apply due to data protection regulations. Anonymized data are, however, available from the corresponding author on reasonable request and with permission of the University Hospital Schleswig-Holstein and the local review board.

## Abbreviations

CI: Confidence interval
EMT: Epithelial–mesenchymal transition
G: Grading
HMGA2: High mobility group A2
IHC: Immunohistochemistry
L: Lymphatic invasion
M: Distant metastasis
N: Nodal spread
PanIN: Pancreatic intraepithelial neoplasm
PDAC: Pancreatic ductal adenocarcinoma
RT: Room temperature
SD: Standard deviation
T: Tumor category
TRAIL: TNF-related apoptosis-inducing ligand
UICC: Union for International Cancer Control
V: Venous invasion
